# On the robustness of the Kelvin probe based potentiometric hydrogen electrode method and its application in characterizing effective hydrogen activity in metal: 5 wt. % Ni cold-rolled ferritic steel as an example

**DOI:** 10.1080/14686996.2019.1687255

**Published:** 2019-11-01

**Authors:** Chun-Hung Wu, Waldemar Krieger, Michael Rohwerder

**Affiliations:** Max-Planck-Institut für Eisenforschung GmbH, Düsseldorf, Germany

**Keywords:** Hydrogen permeation, Davanathan-Starchurski cell, thermal desorption spectroscopy, Kelvin probe, Hydrogen blistering, 212 Surface and interfaces, 106 Metallic materials

## Abstract

Quantitative detection of hydrogen in metal is important in providing a better basis for fundamental investigations of hydrogen embrittlement and hydrogen-related corrosion phenomena. Thermal desorption spectroscopy (TDS) has long been used in characterizing different hydrogen traps inside materials. However, in TDS measurements, the diffusible hydrogen (hydrogen at interstitial sites and weakly bound hydrogen) is usually not detected. The Davanathan-Starchurski permeation technique can cover this shortage. However, for such experiments, the stability of the palladium at the exit side, i.e. in aqueous solution under high potential polarization is an important issue. Alternatively, a Kelvin probe-based (KP-based) potentiometric method developed a few years ago has shown to allow quantitative determination of hydrogen in metal. This method is based on measuring the hydrogen electrode potential on the Pd-coated surface. The aim of this work is to check the reliability of this method and to demonstrate its potential applications in determining the hydrogen amount distributed in both shallow and deep traps in steel. The results reveal that different crystallographic orientation, grain shapes and grain sizes of the deposited palladium film (in the range of variation in this work) do not cause relevant effects on the KP-based hydrogen detection. It is shown in this work that the time lag and permeation rate derived from the permeation curves obtained by this method show a very good reliability and the calculated hydrogen amount shows a good agreement with TDS results. 5 wt.% Ni ferritic steel is used as a model material in this work.

## GRAPHICAL ABSTRACT

**Figure:**
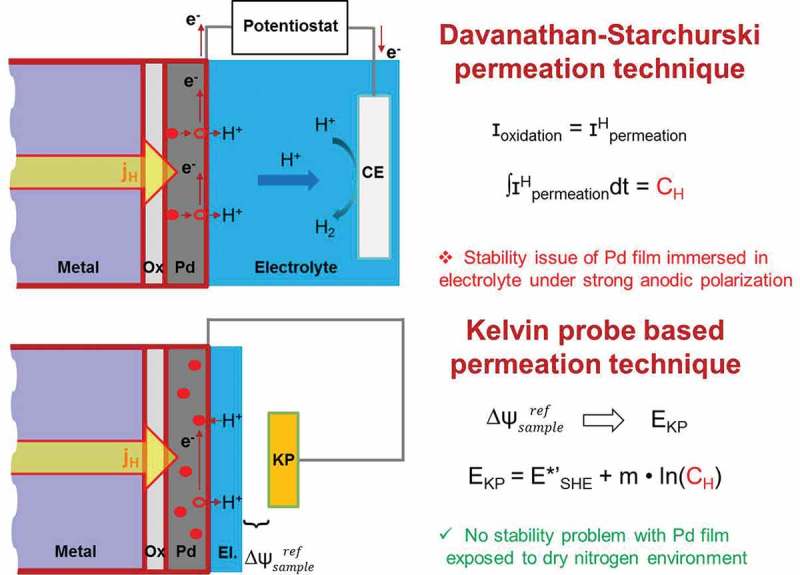

## 1. Introduction

Hydrogen can be a harmful element which causes hydrogen embrittlement (HE) where even tiny amounts of hydrogen (ppm and well below) in a metal might lead to very serious degradation of its mechanical properties [1–3]. Four primary factors are important for causing HE mechanism in steels: the presence of a sufficient amount of diffusible hydrogen, a susceptible microstructure, an applied tensile stress and the ambient temperature in practical applications.

High-strength steels are reported to be more susceptible to HE [4–7] than low-strength steels. One reason is that they have a higher density of defects induced during work hardening process. It is suggested that these defect sites then increase the amount of hydrogen traps in steels and later promote HE damage [7]. On the other hand, low-strength steels are usually subject to a recrystallization process to increase ductility, and most of the cold work-induced defects can be removed. Hence, low-strength steels generally have much lower susceptibility to HE than high-strength steels. Furthermore, ferritic steels have higher susceptibility to HE in comparison to austenitic steels due to the higher mobility of hydrogen in body-centered cubic (bcc) structure than in face-centered cubic (fcc) structure [1,5,8]. Hydrogen may inevitably come from the environment through corrosion or unintentionally come into the material during manufacturing processes (i.e. arc welding, pickling, electrodeposition, etc.). Once hydrogen enters the material, it will be trapped at defect sites (i.e. vacancies, dislocations, voids, etc.) where it will be stronger or weaker bound. Hydrogen at interstitial site and other very weakly bound hydrogen are referred to as diffusible or mobile hydrogen. The location and quantity of mobile hydrogen in metals are two crucial factors in the investigation of HE mechanism and hydrogen storage issues. Concerning HE several mechanisms such as hydrogen-enhanced and strain-induced vacancy formation (HESIV) [9], hydrogen-enhanced decohesion (HEDE) [10,11] and hydrogen-enhanced localized plasticity (HELP) [12,13] have been proposed for single-phase materials. However, things become much more complicated for materials with a complex microstructure, since then local mechanics gain importance over macroscopic mechanical properties. For instance, the HE mechanism of ferritic steels is usually attributed to HELP [14], and HEDE dominates in typical martensitic steels [15]; however, for dual phase (DP) steel, both HELP and HEDE mechanism play important roles due to the easier initiation of localized brittleness and localized plasticity at the interface between two different phases [16].

Once hydrogen is trapped in deep traps of the material, thermal desorption spectroscopy (TDS) can be used to investigate the interaction between hydrogen and local defects (dislocations, voids, etc.) inside the material. The desorption energy of different hydrogen traps can be determined under a given temperature program. Under certain assumptions, the binding energy can be calculated from the desorption energy. In Song’s work [17], he summarized the binding energies of hydrogen at different hydrogen traps in ferritic steels from results presented in the literature. There is often quite significant scatter which may be partially due to the fact that the use of different models (Kissinger, McNabb-Foster and Oriani models) often leads to different results. It can be seen that the binding energy of same hydrogen traps differ in different publications. For example, the binding energy of grain boundaries in ferritic steels is reported to range from 17 to 59 kJ/mol [18–21] and of dislocations ranges from 22 to 59 kJ/mol [18,21–24]. These hydrogen traps can be categorized from zero- to three-dimensional and can be characterized separately by the TDS technique [25]. For example, vacancy is a typical 0D (point) hydrogen trap, and dislocation is a common 1D (linear) hydrogen trap. These two types of defect exist in most materials except perfect crystals. Polycrystalline-structured material possesses an additional 2D (planar) grain-boundary hydrogen traps compared to single-structured material. Besides, the formation of inclusions in the material (e.g. metal carbides, metal oxides, etc.) will also create an additional 2D inclusion–matrix interface hydrogen trap. Voids and blisters are usually categorized as 3D (volume) hydrogen traps.

Although TDS is a powerful technique to investigate the interaction between hydrogen and different hydrogen traps inside the material [17–25], some information is usually lost due to the loss of diffusible hydrogen before the measurement can start. A permeation experiment, on the other hand, is an in situ technique where all hydrogen in material will be kept. The Devanathan-Stachurski (DS) permeation cell invented in 1962 [26] has been widely adapted for investigating hydrogen permeation through metals [27–30]. Usually, a palladium coating is required to be deposited on the exit side sample surface in order to ensure a reliable measurement of the permeation current [31,32]. However, the stability of the palladium layer in aqueous solution under anodic polarization is an important issue during operation. Alternatively, the Kelvin probe based (KP-based) technique provides another way to study hydrogen permeation and has been used for quantitative hydrogen detection [33–36]. One advantage of KP-based setup compared to DS setup is that the palladium film at the detection side is just exposed to dry nitrogen atmosphere, which avoids the instability when immersed in an aqueous solution.

Such an in situ KP-based hydrogen permeation technique was applied in this work to determine the hydrogen amount in metal. 5 wt.% Ni ferritic steel was used in this work as a model material, which careful modification of the microstructures by heat pre-treatments allows to study the contribution of hydrogen amount in different kinds of traps (i.e. voids, vacancies, dislocations, etc.) to total amount of hydrogen in the material. The total hydrogen amount (including hydrogen shallow traps) determined by the hydrogen electrode-based method show good agreement with the results obtained from TDS spectra (only deep traps), just with a small offset value that is proportional to the applied hydrogen activity. This offset value provides additional information of diffusible hydrogen at certain hydrogen activity, which was not accessible by TDS. Combining the results of TDS and hydrogen electrode-based method, the whole picture of which hydrogen amount in each trap contributes to total amount of hydrogen in all kinds of traps in 5 wt.% Ni cold-rolled ferritic steel can be obtained. Considering hydrogen blistering (HB) damage of 5 wt.% Ni cold-rolled ferritic steel, the analysis of HB factors shows that impurity oxide-induced defects (account for ~16%) plays the most important role among all hydrogen traps.

## 2. Experimental procedures

### 2.1. Material

5 wt.% Ni ferritic steel is used as model material in this work, and main chemical composition (>0.002 wt.%) is shown in Table 1. 12 kg of a model ferritic alloy with 5 wt.% Ni was molten at 1400°C for 2 h in an induction furnace (IM) and was cast into a rectangular 200 × 40 mm^2^ copper mold, which was fabricated in house. Five kinds of heat treatment process were performed on this 5 wt.% Ni ferritic steel in order to achieve different microstructures of each sample. The annealed sample (A) was directly obtained from the cast 5 wt.% Ni ferritic steel. The cold-rolled sample CR (IM) was prepared by mechanical rolling of the annealed sample with a subsequent thickness reduction from 10 mm to 1.5 mm. Another cold-rolled sample referred as CR (AM) was produced by heating the cast 5 wt.% Ni ferritic steel in an arc melting furnace (AM) through same annealing and cold work process. The recovered sample referred as CR (IM) – 300°C for 24 h was made by heating the cold-rolled sample to CR (IM) – 300°C where the temperature is high enough for recovery but not enough for recrystallization process and kept for 24 h. The recrystallized sample (RK) was prepared by heating the cold-rolled sample CR (IM) up to 500°C for 24 h. Some key mechanical properties and material characterization of the three steel samples (A, CR, RK) are presented in Table 2.

**Table 1.** Main chemical compositions of 5 wt.% Ni ferritic steel [25].

Alloy elements	C	Ni	Cu	Mn	O	Cr	S	N
Weight percentage (wt.%)	0.0026	5.05	0.047	0.0373	0.0307	0.0076	0.0032	0.0028

**Table 2.** Key mechanical properties and material characterizations of the 3 different pre-treated 5 wt.% Ni ferritic steels [25].

5 wt.% Ni ferritic steel	Hardness (HV5)	Strength (MPa)	Grain size (μm)	Dislocation density (m^−2^)
Annealed (A)	118 ± 1	355 ± 7	41 ± 5	7.38 × 10^14^
Cold-rolled (CR)	249 ± 9	840 ± 22	–	2.14 × 10^16^
Recrystallized (RK)	128 ± 2	397 ± 31	15 ± 2	1.12 × 10^14^

### 2.2. Kelvin probe-based hydrogen permeation experiment

The five differently heat-treated steel samples used in this work were mechanically polished to remove the thick oxide layer and organic contaminations from the surface present in the as received conditions. The polished samples were then immersed in ethanol in an ultrasonic cleaner for 10 min to clean the surface. The samples taken out from the ultrasonic cleaner were rinsed with CCl_4_ to remove remaining organic contaminants adsorbed on the surface and then dried with 45°C hot air. Afterwards, a 100 nm Pd thin film is always deposited on one side by physical vapor deposition (PVD, Leybold Univex 450), denoted as ‘old’ PVD system. It is working as the exit side sensor for the hydrogen permeation study [37–39]. In the experiments for Pd layer characterization, some Pd thin films were prepared with another PVD system (custom-made multi-chamber cluster by BESTECTM), denoted as ‘new’ PVD system. The in situ experimental setup used in this study was first introduced in Evers et al.’s work [37]. An electrochemical cell is positioned on top of the sample to control hydrogen loading at the entry side. A KP is positioned below the sample to measure the hydrogen-induced potential change at the exit side [37,38]. The exit side chamber is always purged with dry (0 rh%) nitrogen gas during the measurements. The tip of the KP microscope is made from a Ni80Cr20 alloy wire by electro-polishing [40], with a diameter of 100 µm. The working electrode area (hydrogen charged area) is 12.57 mm^2^ (defined by an O-ring with a diameter 4 mm). A 25 µm thick pure Pt foil (99.99%, temper: as rolled) used as a counter-electrode (CE) was purchased from Goodfellow GmbH and a commercial silver/silver chloride electrode (Ag/AgCl, length 12.5 cm) with 3 mol/L KCl filling used as a reference electrode (RE) was purchased from Metrohm. The area of the CE is 12.5 cm^2^. All the chemicals used in this work were purchased from VWR international GmbH. A potentiostat/galvanostat power supply (PGU 10V-1A-E) purchased from IPS Elektroniklabor GmbH & Co. KG was used for the electrochemical analysis. Hydrogen was loaded electrochemically from the entry side steel surface and was measured at the exit side Pd-coated surface by KP. Different from Devanathan-Stachurski permeation cell where hydrogen oxidation current is measured, in this case, the hydrogen electrode potential in dry N_2_ atmosphere is measured [37–39].

### 2.3. Hydrogen blistering experiment

In this experiment four steel samples: CR (IM), CR (AM), CR (IM) – 300°C for 24 h and RK were first ground with sand paper (SiC P1000) to remove thick oxides and contaminations presented in as received condition. Afterwards, they were rinsed with ethanol and dried with 45°C hot air. The steel samples were pre-charged with hydrogen in an electrochemical cell for 1 h at different hydrogen activities and later taken to optical microscope (OM) and scanning electron microscope (SEM) to observe surface morphologies and cross-sectional views of hydrogen pre-loaded region. Energy-dispersive X-ray spectroscopy (EDX) mapping was used to analyze the chemical composition of impurity oxides. The materials and working area of electrodes (CE, RE, WE) used in this experiment are same as described in Section 2.2. The charging electrolyte is 0.1 M sulfuric acid and thiourea (THS) was added in some cases to increase hydrogen activity on charging side surface.

## 3. Results and discussion

### 3.1. Theoretical background: potentiometric hydrogen electrode method

**Figure 1.**
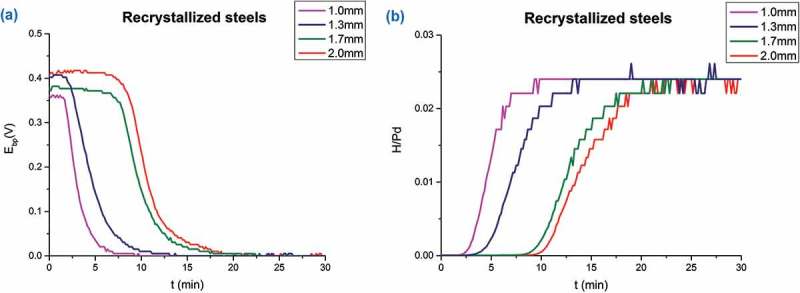
(a) Potentials measured by Kelvin probe at the exit 100 nm Pd thin film-coated side during hydrogen loading of RK steels with different thicknesses. (b) Permeation curves obtained by transferring (a) with Nernst equation (Equation 3). All samples are loaded with hydrogen under same conditions at the entry cell (0.1 M H_2_SO_4_ + 0.2 mM thiourea, galvanostat: −1.2 mA/cm^2^).

**Figure 2.**
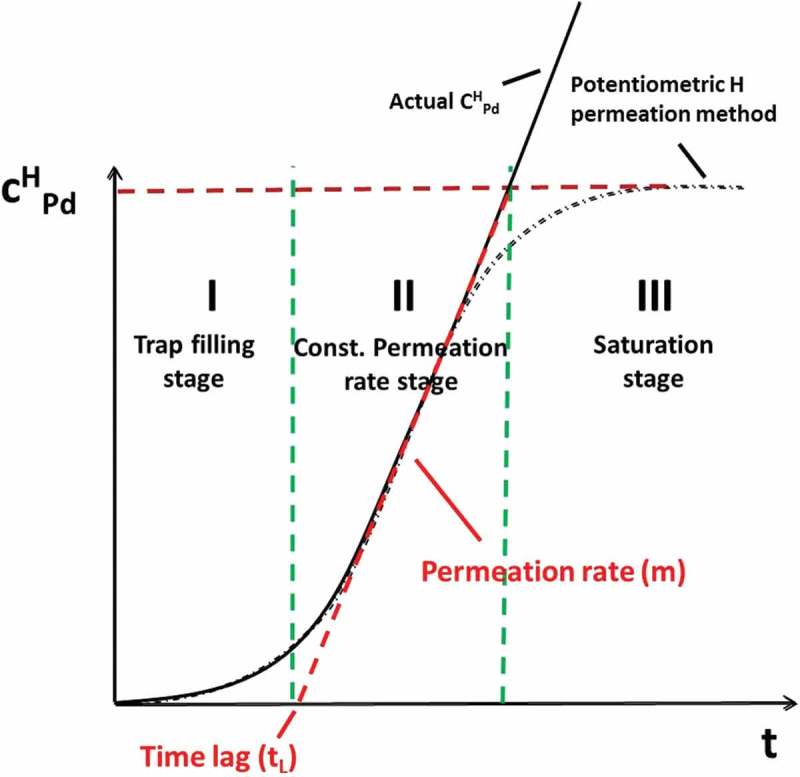
A typical permeation curve obtained by potentiometric hydrogen electrode method based on measuring hydrogen electrode potential of a thin Pd detection layer.

**Figure 3.**
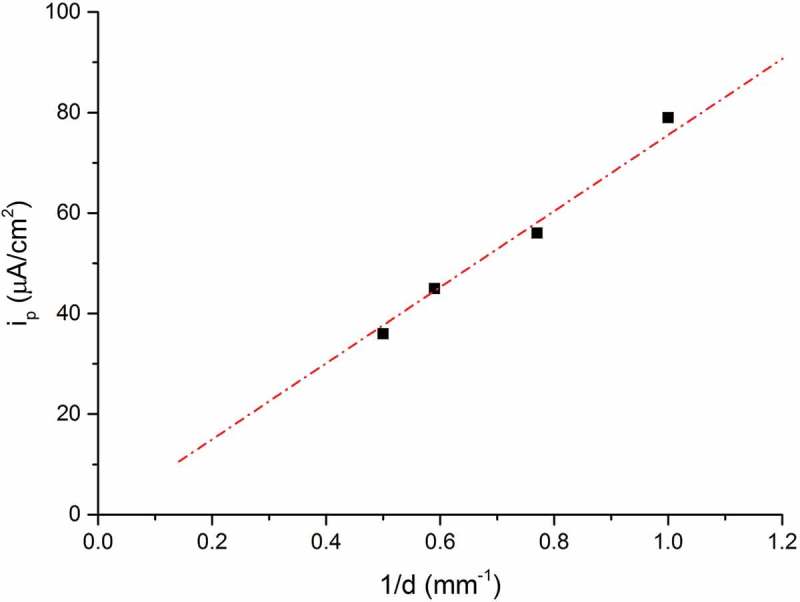
Plot of permeation current (*i*_p_) versus the inverse of thickness (1/d). (Data from Figure 1).

The KP-based potentiometric method for the detection of hydrogen was so far mainly used for hydrogen mapping [37–39,41–43]. However, it was already shown that this method in principle can be also used for permeation measurements [37,38]. In the absence of oxygen, the surface potential measured on Pd-containing hydrogen by KP is comparable to measuring the hydrogen electrode potential in bulk solution and follows the Nernst equation [37,38]. From the Nernst equation, it is known that the hydrogen electrode potential is governed by the proton activity in the electrolyte and the square root of partial pressure of hydrogen gas (i.e. the equilibrium of the hydrogen electrode). In our case, it is convenient that the partial pressure term is replaced by the hydrogen activity in the Pd layer. Based on prior work of Evers et al. [37,38], if we use the hydrogen concentration instead of hydrogen activity in Pd, we can get the following relation between measured potential value (E) and hydrogen concentration in Pd (cH_Pd_).

(1) $$E={E^{{*}^{,}}}_{SHE}+m∙ln\left({cH}_{Pd}\right)$$

where E^*’^_SHE_ denotes to standard hydrogen electrode potential at the pH in the surface layer (which we do not know yet). The theoretical value of the slope (m) should be −59 mV/decade of activity or concentration at room temperature, but it was also reported in the literature [44] that different microstructures of Pd cause a deviation from this theoretical value for the concentration dependence, due to the non-equality of activity and concentration. Single-crystal structure of Pd gives a value close to the theory, while structures with high dislocation density or polycrystallinity will cause a deviation from the theoretical value. In our case for a thin PVD deposited Pd film of 100 nm thickness, m = −130 mV/decade of hydrogen concentration was measured [37,38], which is proposed to be due to the nano-crystalline structure of the Pd thin film deposited by PVD and its interface with the steel, acting both as trap sites, which leads to a deviation between activity and concentration in the Pd. For convenience we set the binary phase potential as 0 V (also referred to as a reference potential *E*_bp_ in this study). The binary phase potential is determined by the potential being pinned with increasing hydrogen in Pd, since above about 2.4% atomic ratio of H in Pd at room temperature (RT) hydrogen in Pd starts to form β-H-Pd. As long as α-H-Pd and β-H-Pd coexist in parallel, the concentration of hydrogen is pinned at a critical value, fixing the potential (see [37]). With known slope −130 mV/decade and the critical concentration value of hydrogen (H/Pd: 0.024 at RT at onset of *E*_bp_, where palladium hydride starts to form in Pd), we can directly calculate the concentration of hydrogen in Pd from the potential. In other words, one can easily transfer the potential data into a permeation curve (dE/dt to dc/dt), and this method we call hydrogen electrode-based potentiometric method. Figure 1(a) shows the potential measured by the KP (referenced to binary phase potential) of RK samples with different thickness. The slight difference in the initial potential value might probably be due to the existence of hydrogen in the steel sample before Pd deposition which upon entering into the Pd layer results in slightly lower initial potential (note that due to the logarithmic dependence of E on C the corresponding H activities at this initial period are very low). Figure 1(b) shows the transformed permeation curves by using Equation (3) on the data from Figure 1(a). A sketch of a typical permeation curve with this approach is shown in Figure 2, depicting three main stages: trap filling, constant permeation rate and saturation (binary phase: α-H-Pd and β-H-Pd) stage. In the initial trap filling stage, it takes time for hydrogen to fill up all kinds of traps (vacancies, precipitates, dislocations, etc.) and to reach the equilibrium concentration in the lattice corresponding to the applied hydrogen activity (a_H_) inside the material. Finally, a constant gradient of hydrogen concentration through the whole sample is established, resulting in a constant permeation rate stage (steady-state permeation rate) where a linear increase of hydrogen concentration in the Pd is observed. Once the hydrogen concentration in Pd reaches the critical value, which is about 2.4% atomic ratio in room temperature, it will then come to the binary phase region (saturation stage of the potential). There are two important values one can obtain from such a permeation curve: the slope and its intercept with the *x*-axis. From the slope, one can obtain the final steady-state hydrogen permeation rate, while from the intercept with the *x*-axis the time lag (*t*_L_) the time required for hydrogen to fill up all kind of traps inside the material can be determined. As is shown in Figure 1(b), the thinnest sample (1 mm) has the highest permeation rate (highest slope). This is because the hydrogen permeation rate is proportional to the inverse of the sample thickness. It can also be found out that under same charging condition, the thickest sample has the longest trap filling stage (longest time lag *t*_L_) which indicates the thickest sample has the highest amount of hydrogen traps, which should be proportional to the sample thickness. The linear relationship between permeation currents (*i*_p_) and the inverse of sample thickness (1/d) is presented in Figure 3. This is in good agreement with literature result [45] that under same charging condition at entry side, the permeation current measured at the exit side is linearly proportional to the inverse of sample thickness.

### 3.2. The effects of Pd thin film properties and Pd/substrate interfaces on obtained permeation results

**Figure 4.**
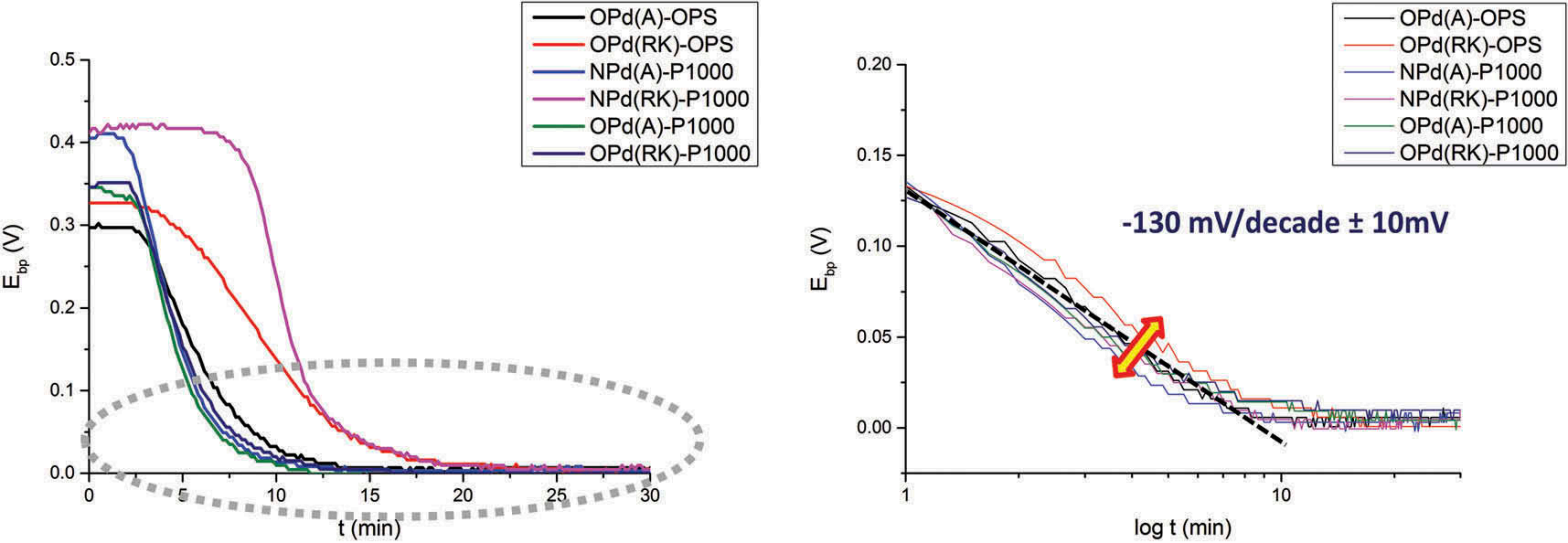
Left: hydrogen electrode potential measured by Kelvin probe at Pd-coated exit side; Right: plot of E vs. log t with time lag subtracted from raw permeation data: in the gray dash circle of left graph. (OPd – Pd thin film from old PVD system, NPd – Pd thin film from new PVD system, OPS – sample polished with OP-S, 0.025 µm, P1000 – sample ground with sand paper SiC P1000, A – annealed XFeNi5 steel, RK – recrystallized XFeNi5 steel). All samples with same thickness (1 mm) are loaded with hydrogen under same conditions at the entry cell (0.1 M H_2_SO_4_ + 0.2 mM thiourea, galvanostat: −1.2 mA/cm^2^).

**Figure 5.**
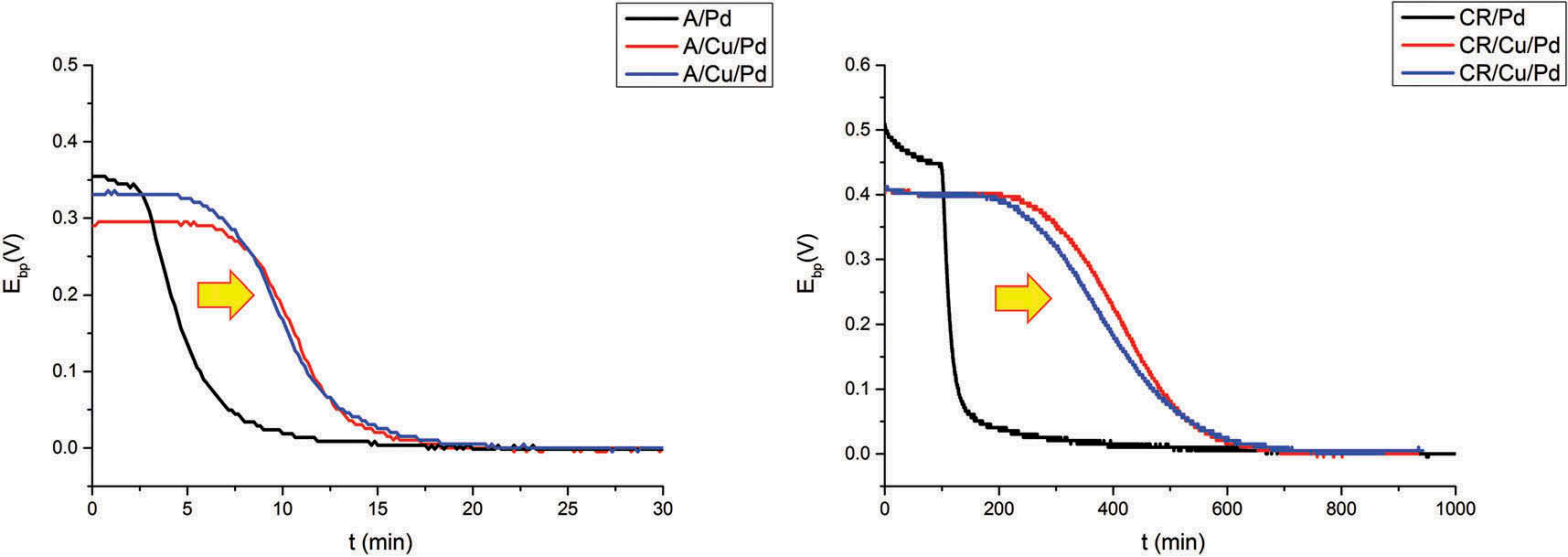
The influence of additional barrier layer (200 nm Cu) on hydrogen electrode potential measured by Kelvin probe at Pd-coated exit side. Left: with and without Cu layer between annealed (A) steel and Pd coating; Right: with and without Cu layer between cold-rolled (CR) steel and Pd coating. All samples with same thickness (1.35 mm) are loaded with hydrogen under same conditions at the entry cell (0.1 M H_2_SO_4_ + 0.2 mM thiourea, galvanostat: −1.2 mA/cm^2^).

**Figure 6.**
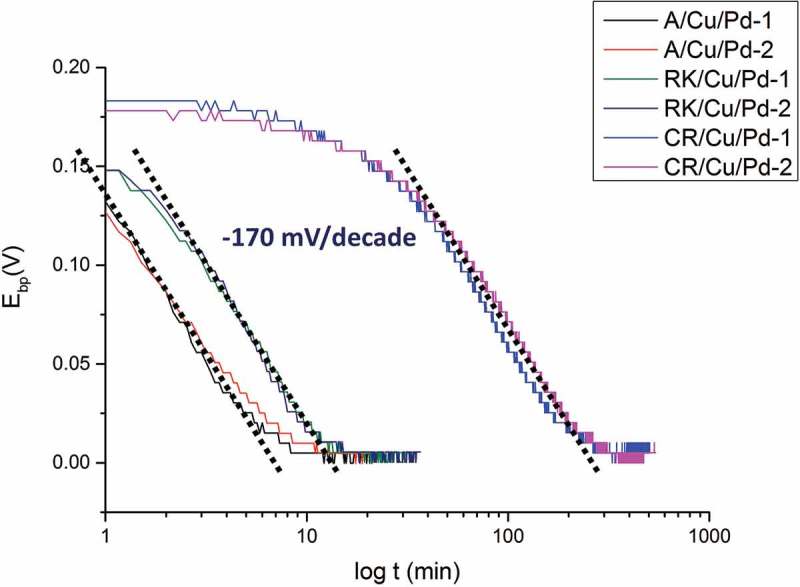
Plot of E vs. log t with time lag subtracted from raw permeation data. All steel samples (A, RK, CR/Cu/Pd; 1&2 are labelled for sample 1 and sample 2) are charged with hydrogen for entry steel side under same conditions with same specimen thickness (1.35 mm). All samples with same thickness (1.35 mm) are loaded with hydrogen under same conditions at the entry cell (0.1 M H_2_SO_4_ + 0.2 mM thiourea, galvanostat: −1.2 mA/cm^2^).

Figure 4 shows the raw hydrogen permeation results obtained on different kinds of samples (different pre-treatments, PVD system) measured by KP. It can be seen that in the initial stage (time before break through) the curves differ a lot from each other (with different starting potentials, break down times, etc.) while in the region close to binary phase potential (*E*_bp_ = 0) all of them show similar behaviors. Different starting potentials might be caused by different origin of the hydrogen content inside the steel which already initially enters Pd layer and results in lower starting potential. The starting potentials of Pd in dry (0 rh%) pure nitrogen environment usually range from 0.3 V to 0.5 V against binary phase potential (*E*_bp_) depending on sample preparations. Different breakthrough times (when the potential starts to decrease sharply) are correlated to different total amount of all kinds of hydrogen traps in the steel (i.e. voids, vacancies, dislocations, etc.). It can be observed in Figure 4 (left) that A has a shorter break down time than RK which is due to the lower trap amount in A than in RK. Figure 4 (right) shows a closer view of the final stage in which the hydrogen concentration gradients are already established through each sample. Despite the differences observable in the initial stage, all samples exhibited similar behavior with very slight differences in the last stage (−130 mV/decade with an error bar ±10 mV). The results indicate that the PVD systems (old and new PVD system) investigated here and surface roughness (P1000 and OPS) will not greatly influence the potential dependence on H concentration, i.e. the Pd films show little effects on the curves. In addition, the case that a barrier layer formed at the interface of steel substrate and Pd thin film layer is also investigated. A 200 nm copper thin film was deposited by old PVD system, acting as barrier layer since hydrogen diffusion in Cu is much lower than in Fe. It is reported in the literature that at room temperature (298 K) hydrogen diffusivity in fcc structure copper is (~10^−13^ m^2^/s [46]) while in bcc structure α-iron is (~10^−8^ m^2^/s [47]). The aim is to simulate the situation when before Pd coating PVD deposition the sample has already been covered by a contaminated layer or thick oxide which seriously impedes hydrogen to diffuse through. The effects of this extra barrier layer can be seen in Figure 5. It can be seen that the samples with extra 200 nm copper layer show obviously extended time before break through than samples without copper layer under same hydrogen charging condition at the entry side. Besides, A,CR/Cu/Pd samples also exhibit relatively stable potential plateaus in the initial stage compared with A,CR/Pd samples. It should be noted here that the decrease of potential is due to the electrochemical reaction of hydrogen at the Pd surface where hydrogen give electrons to Pd and forms protons [37]. A sharp decrease of potential after breakthrough time is caused by huge amount of hydrogen coming to Pd after saturation (all traps are fully occupied with hydrogen). The delay of breakthrough time might be caused by increasing hydrogen traps or slower diffusion rates. In this case, increasing hydrogen traps in the additional Cu layer thin layer (200 nm) is almost negligible in comparison to steel (1 mm), so the difference is assumed to be mainly due to lower diffusivity of hydrogen in Cu layer (5 magnitude lower than in α-Fe). Besides, small amount of hydrogen might still come to Pd layer before saturation and result in slight decrease of potential (A/Pd, CR/Pd). With the application of a barrier layer (A/Cu/Pd, CR/Cu/Pd), this becomes impossible because of relatively slow diffusion process. Hence, it shows a plateau in the initial stage (no hydrogen enters Pd layer). As soon as all trap sites are filled both cold-rolled and annealed samples show almost no differences in the permeation kinetics. Only slightly deviating slopes of −130 mV/decade ±10 mV were measured (Figure 4, right graph) regarding the effects of Pd thin film properties and surface roughness. Steeper slopes of −170 mV/decade (see Figure 6) measured on A, RK, CR/Cu/Pd samples indicate there are more trap sites (probably at Cu–Pd interface) generated than A, RK, CR/Pd samples leading to much more serious deviation of the theoretical value (−59 mV/decade). The overall comparisons of different Pd sources (Pd thin film prepared from old and new PVD systems), surface treatments (samples ground with SiC sand paper P1000 and polished with OP-S 0.025 µm particles), different substrates (Pd thin films on glass and steel) and extra barrier layer (with and without Cu layer between steel and Pd layer) are listed in Table 3. Despite the differences in surface roughness, grain sizes and crystal planes, they do not cause large differences in the slope of E vs. log t (±10 mV). The only effect lies in the case with the formation of an additional barrier layer formed at substrate/Pd interface, which induces more hydrogen trap sites and leads to severe deviation of the slope (−170 mV/decade).

**Table 3.** Overview of the Pd thin film properties under different conditions (PVD systems, surface roughness, substrates, barrier layer) and their influence on Pd film and the slope is according to Equation (1) for the hydrogen electrode-based method.

	Pd (old PVD) on steel (P1000 grinding)	Pd (old PVD) on steel (OPS polishing)	Pd (new PVD) on steel (P1000 grinding)	Pd (old PVD) on glass substrate	Pd (old PVD) on Cu/steel (OPS polishing)
RMS roughness	7.6n m	53.83 nm	7.6 nm	-	53.83 nm
Mean Pd grain size	15 nm	15 nm	5 nm	20 nm	15 nm
Grain shape (top view)	Sphere	Sphere	Sphere	Elongated	Sphere
Preferred orientation	(111) (200) (220)	(111) (200) (220)	(111)	(111) (200) (220)	(111) (200) (220)
E vs. log t slope	−130 ± 10 mV/decade	−130 ± 10 mV/decade	−130 ± 10 mV/decade	−130 ± 10 mV/decade [37]	−170 ± 5 mV/decade

### 3.3. Time lags (t_L_) and permeation rate (i_p_)

A summary of steady-state permeation rates and time lags of the three steel samples (A, RK, CR (IM); thickness 1 mm) under same charging condition (galvanostat: −0.4 mA/cm^2^) with different thiourea (THS) concentrations in 0.1 M sulfuric acid are listed in Table 4.

**Table 4.** The permeation rates and time lags obtained with the hydrogen electrode-based potentiometric method (different concentration of thiourea (THS) in 0.1 M H_2_SO_4_, galvanostat: −2 mA/cm^2^, sample thickness: 1 mm).

c(THS)_el_		0 mM THS	0.2 mM THS	2 mM THS	20 mM THS
A	*i*_p_ (μA/cm^2^)	4.9 ± 0.4	58 ± 3	74 ± 5	84 ± 6
	*t*_L_ (s)	1161 ± 112	174 ± 35	157 ± 42	147 ± 30
CR	*i*_p_ (μA/cm^2^)	4.3 ± 0.3	22 ± 19	27 ± 23	32 ± 27
	*t*_L_ (s)	12,500 ± 498	4254 ± 2466	2066 ± 1174	1203 ± 612
RK	*i*_p_ (μA/cm^2^)	4.5 ± 0.6	49 ± 4	64 ± 6	74 ± 6
	*t*_L_ (s)	1286 ± 185	266 ± 38	230 ± 36	211 ± 47

#### 3.3.1. Time lags (t_L_) from permeation curve

It is shown in Table 4 that in the case without addition of THS, the permeation rates of three steel samples lie in the similar range of 4~5 µA/cm^2^ while the time lags of CR (IM) is much higher than A and RK. The similar permeation rate of three steel samples is not so surprising here, since the constant permeation rate is expected after hydrogen has filled up all the hydrogen traps in the material and this is in good agreement, e.g. with Riecke et al.’s work [48]. The difference in time lag of the three steel samples strongly reflects the different amount of hydrogen traps in each sample. The CR (IM) sample has the largest time lag in comparison to A and RK, while the difference between A and RK is relatively small. These three steel samples (5 wt.% Ni ferritic steel) all have the same composition. The cold work-induced defects are supposed to be the main feature of hydrogen traps. It is shown in Table 2 that CR (IM) steel has much higher dislocation densities than A and RK steels. This is consistent with hydrogen permeation experiments showing that highest amount of defects in CR (IM) steel results in largest time lag. The difference of time lags between A and RK is difficult to elucidate in detail. In some experiments, larger time lags are measured on A samples while in some cases, larger time lags are measured on RK samples (i.e. this is certainly due to scatters in the trap density), so it is assumed that the amount of hydrogen in equilibrium with the applied potential is similar.

#### 3.3.2. Permeation rate (i_p_) from permeation curve

THS is a well-known promoter used to increase hydrogen uptake into iron and steels [45,49–53], and the mechanism is due to the inhibition of hydrogen recombination reaction [50–52]. It can be seen in Table 4 the final steady-state permeation rate of A and RK greatly increases about 10 times with addition of 0.2 mM THS in the electrolyte and shows a moderate further increase in 2 mM THS (1.3 times) and 20 mM THS (1.2 times). The results show a similar tendency with the work of Dull and Nobe [49], where the effects of thiourea and triazoles on steady-state hydrogen penetration rates into iron were investigated. It is shown in their work that the steady-state hydrogen penetration in iron under the same charging condition (−2 mA/cm^2^) as in this work without THS in 1 N sulfuric acid is about 8 µA/cm^2^ (sample thickness: 1 mm). With addition of 0.2 mM THS, the permeation rate increases to 70 µA/cm^2^, and becomes 90 µA/cm^2^ and 120 µA/cm^2^ with further 10 times THS concentration (2 mM THS, 20 mM THS). The higher hydrogen permeation rate obtained in Dull’s work than in this study might be probably due to higher sulfuric acid concentration used in his experiments (1 N sulfuric acid, in our experiment 0.1 M) and/or different materials. Unlike the sharp increase observed for in A and RK, the increase of permeation rate with 0.2 mM THS in CR (IM) steels is lower (only five times increase in average) and shows a relatively bigger error bar.

According to the work of Dafft et al. [45], measured values well below the expected permeation rates and low reproducibility after the addition of promoters into the solution can be attributed to the following three causes:

1. Decrease of concentration of the substance that promotes hydrogenation.
2. Reduction of the stationary diffusion within the membrane by hydrogen-induced cracks (volume effect).
3. Obstruction of hydrogen absorption through deposits formed on iron during cathodic polarization in the presence of promoters.

In this work, it was found in the surface images obtained by SEM that many blisters formed on CR (IM) surface after hydrogen permeation experiments (with 0.2 mM and higher concentration of THS), so the point 2 is the most possible explanation that leads to the unusually low permeation rates of CR (IM) steels in our case. Therefore, the large deviation of permeation rate (*i*_p_) and time lag (*t*_L_) obtained for the CR (IM) steel (with 0.2 mM, 2 mM, 20 mM THS in the electrolyte) is attributed to blister formation, since blisters form randomly and our measurements are quite locally (tip diameter is just 100 μm). When the diffusion path of hydrogen in the steel is blocked by the formation of blisters an extremely low permeation rate (*i*_p_) is measured and if not, a permeation rate (*i*_p_) close to the cases of A and RK was measured. The time lag (*t*_L_) reflects the time required to fill the traps in the steel before the constant, final permeation rate is reached (see Figure 2). Therefore, when the diffusion of hydrogen is hindered by blister formation (decrease of permeation rate), the corresponding time lag will increase. This conclusion is supported by close standard deviation (*i*_p_ and *t*_L_) of A, RK and CR when charging at low hydrogen activity (no THS, i.e. when there is no blister formation). The permeation data (*i*_p_ and *t*_L_) obtained by the hydrogen electrode-based potentiometric method show reliable and comparable results obtained with other techniques reported in literature [45,48–53]. The permeation rate (*i*_p_) can be used to study hydrogen uptake into the material surface and it will be shown that the time lag (*t*_L_) can be used to get an insight into total amount of hydrogen traps in the material.

### 3.4. Characterization of different hydrogen traps in 5 wt.% Ni CR(IM) ferritic steel

#### 3.4.1. Diffusible hydrogen and trapped hydrogen

It is quite difficult to determine how the hydrogen in a material distributed at the different trap sites and what the exact nature of these trap sites really is. Often TDS is applied which can provide already a good overview about the number of the different kinds of main trap sites. As will be shown in the following the contribution of each kind of hydrogen trap site to the total hydrogen amount in the material can also be elucidated by a step-by-step modifications of the steel samples. Hydrogen in interstitial sites and reversible (shallow) traps is referred as diffusible hydrogen while hydrogen in deep traps is referred as trapped hydrogen. The total diffusible hydrogen amount can be obtained from permeation measurements with the help of the following equations [54]:

*Diffusible hydrogen – hydrogen in shallow traps*

(1) $$C_{Hd,0}=\frac{i_{P}*L}{F*D}$$

Here C_Hd,0_ is the concentration of diffusible hydrogen at the entry side of the sample (mol H per unit volume), *i*_p_ is the steady-state per rate, L is the sample thickness, F is Faraday constant, D is diffusion coefficient of hydrogen in the steel. The effective (or apparent) hydrogen diffusivity D_eff_ [54–58] is usually adapted in this case, which can be obtained by following equation:

(2) $$D_{eff}=\frac{L^{2}}{6*t_{L}}$$

where *t*_L_ is the lag time, defined as 0.63 times the steady-state value [58], L corresponds to the sample thickness.

Boes and Züchner compared the time lag method under galvanostatic and potentiostatic hydrogen loading, and the diffusion coefficient D’ obtained under galvanostatic charging is obtained according to [56]:

(3) $$D^{\;,}=\frac{L^{2}}{2*t_{L}}$$

The lattice diffusion coefficient of diffusible hydrogen (*D*_L_) in pure iron at room temperature is reported in the literature in the range between 5~9.6 × 10^−5^ cm^2^ s^−1^ [54,58,59]. In a standard permeation experiment, the exit side diffusible hydrogen concentration is kept at 0. With known *D*_L_ (7.5 × 10^−5^ cm^2^ s^−1^ is used in this work for calculation) the steady-state diffusible hydrogen amount (mol H per unit area) in the sample can be calculated by following equation:

(4) $$q_{Hd,0}=\frac{C_{Hd,0}*L}{2}$$

and according to (1) and (3)

(5) $$q_{Hd,0}=\frac{i_{P}*t_{L}}{F}$$

the total diffusible hydrogen concentration is shown (weight ppm):

(6) $$C_{diff.\;H}=\frac{q_{Hd,0}*A_{s}*M_{H}}{W_{steel}}*10^{6}$$

where A_s_ is total surface area (cm^2^), M_H_ is the molar mass of hydrogen (g/mol) and W_steel_ is the weight of steel samples (g).

##### 3.4.1.1. Trapped hydrogen – hydrogen in deep traps

According to Stevens and Bernstein’s work, the time-lag method can be used to study the effect of trapping on hydrogen diffusion. With saturable traps in the material, the total time lag can be expressed as below [57]:

(7) $$t_{lag}=t_{L}\left\{1+\frac{3*\alpha }{\beta }+\frac{6*\alpha }{\beta^{2}}+\frac{6*\alpha }{\beta^{3}}\left(1+\beta \right)ln\left(1+\beta \right)\right\}$$

here *t*_L_ is the time lag for pure lattice diffusion, and α, β are defined by:

$$\alpha =N_{T}\frac{k}{p}$$

$$\beta =C_{o}\frac{k}{p}$$

where N_T_ is the total trap density (mol H per unit volume), C_o_ is the input concentration at entry side (mol H per unit volume), and k, p denote to statistical trapping and detrapping parameters.

When C_o_ is very large (high hydrogen activity at entry side), Equation (6) can be simplified to:

*t_lag_ ≅* $t_{L}\left\{1+\frac{3*\alpha }{\beta }\right\}\;$*=*$\;t_{L}\left\{1+\frac{3*N_{T}}{C_{o}}\right\}$

C_o_ is taken from Equation (1), and the diffusion coefficient is replaced by effective diffusivity (D’), then total trap density can be obtained as below:

(8) $$N_{T}=\frac{3*i_{p}*t_{Trap}}{2*F*L}$$

the term (*t*_lag_ – *t*_L_) is substituted by the time lag caused by deep traps (*t*_Trap_), and the total trap hydrogen concentration is shown (weight ppm):

(9) $$C_{Trap\;H}=\frac{N_{T}*V*M_{H}}{W_{steel}}*10^{6}$$

#### 3.4.2. Comparison with TDS data

**Figure 7.**
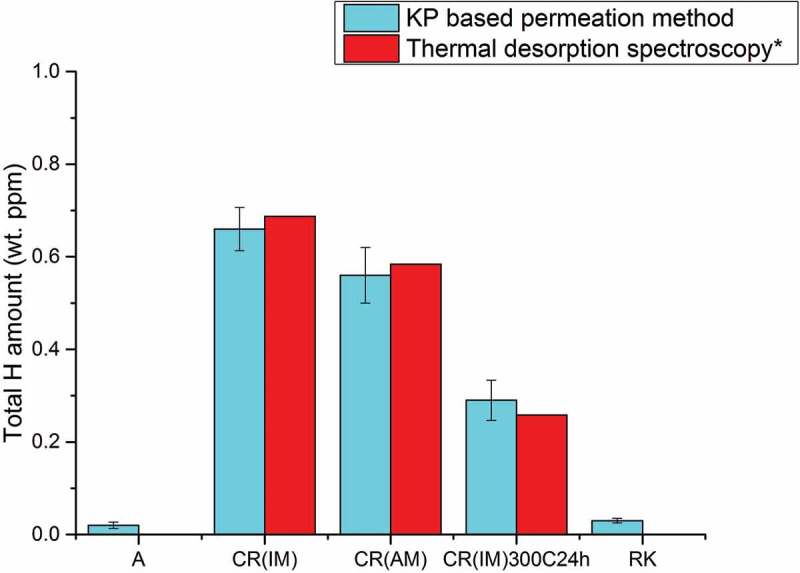
A comparison of total hydrogen amount in different modified 5 wt.% Ni ferritic steels obtained by hydrogen electrode-based method and TDS technique (*TDS data come from literature [25]). All samples with same thickness (1 mm) are loaded with hydrogen under same conditions at the entry cell (0.1 M H_2_SO_4_ + 0.02 mM thiourea, galvanostat: −2 mA/cm^2^; a_H_: 60).

**Figure 8.**
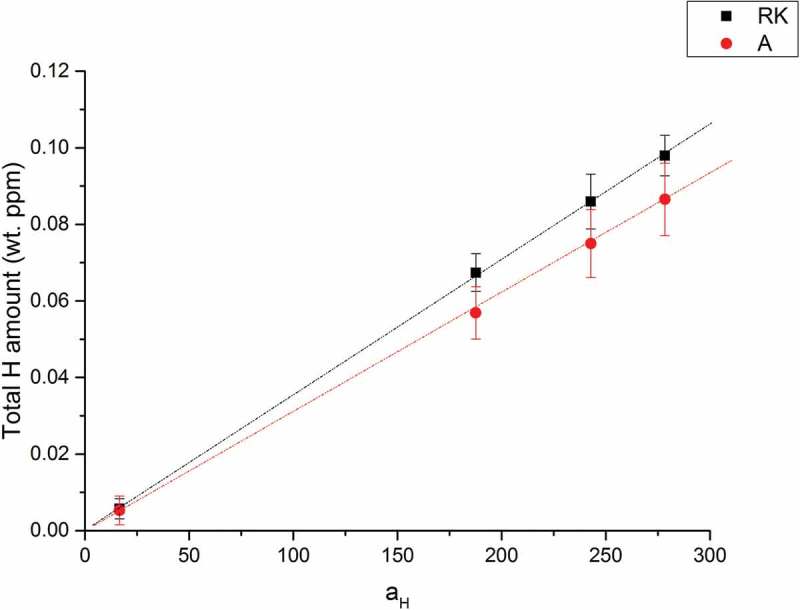
Plot of total hydrogen amount versus entry side hydrogen activity of recrystallized (RK) and annealed (A) steels. The hydrogen activity (a_H_) is assumed to be similar to Dafft et al.’s work on pure iron [45].

Figure 7 shows the total hydrogen amount calculated from KP-based permeation results using Equations (6) and (9) and TDS results [25]. Since A and RK do not have deep traps [25], the hydrogen concentration are calculated from Equation (6). CR (IM), CR (AM) and CR (IM) – 300°C for 24 h are calculated from Equation (9). The values from TDS results are calculated from the integration of the area below the TDS spectra. It can be seen that for hydrogen in deep traps, the calculation of the results from the KP-based permeation method show good agreement with the TDS data. This also indicates that the trapped hydrogen was indeed quantitatively remaining in the material after the hydrogen loading process and was later fully detected by the TDS measurement. However, diffusible hydrogen was not accessible by TDS, which was proven by flat spectra obtained on A and RK samples [25]. Therefore, the total hydrogen amount calculated from the TDS spectra of A and RK is 0. In situ permeation experiment is capable to measure the diffusible hydrogen (see Figure 7). Since the amount of diffusible hydrogen is proportional to the applied hydrogen activities at the entry side (hydrogen in deep traps does not change with hydrogen activity, once filled), the amount of diffusible hydrogen at ambient environment (a_H_: 1) can be obtained by plotting hydrogen amount with different applied hydrogen activity at entry side (see Figure 8). It can be seen that at a_H_ = 1 the concentration is about 0.00035 wt. ppm which is close to the hydrogen solubility of diffusible hydrogen reported in the literature in α-iron at a_H_ = 1 without deep traps (0.00039 × wt. ppm [48]).

#### 3.4.3. Hydrogen trap type in 5 wt.% Ni cold-rolled ferritic steel – CR (IM)

**Figure 9.**
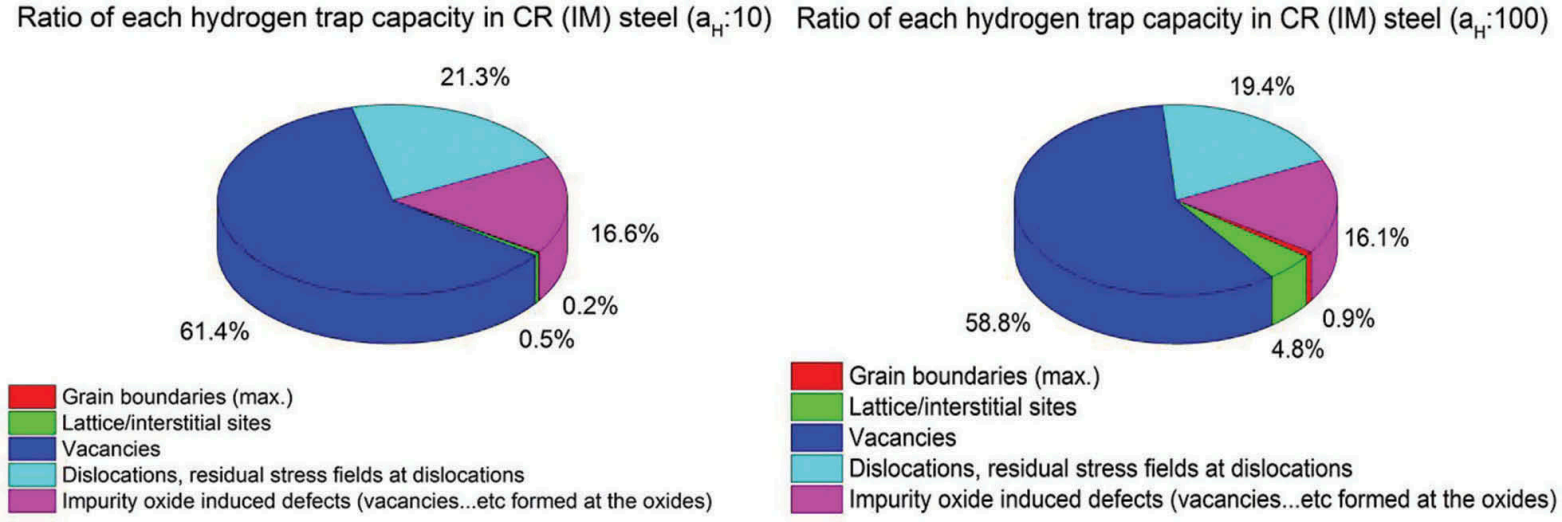
A pie chart describing the contributions of hydrogen in each individual kind of trap to total amount of hydrogen in 5 wt.% Ni cold-rolled CR (IM) steel at different hydrogen activities (a_H_: 10 & 100).

In the following CR (IM) is taken as a reference sample to study the contributions of different kind of defects to the total amount of hydrogen traps. The other steel samples (same chemical composition, Table 1) were treated with different heat treatments in order to remove certain kind of hydrogen trap (i.e. CR(IM) – 300°C for 24 h: annealing at 300°C for 24 h to remove most vacancies, CR (AM): arc welding instead of induction welding to reduce impurity contamination). The decrease of total hydrogen amount calculated in each sample (A, CR(AM), CR(IM) – 300°C for 24 h, RK) in comparison to CR (IM) is then attributed to the contribution of one or more certain types of hydrogen traps in the CR. Since there was no hydrogen in deep traps measured by TDS, the calculated total hydrogen amount of A is attributed to hydrogen at interstitial sites and of RK is hydrogen at interstitial sites and grain boundaries. Hydrogen at interstitial sites and grain boundaries are referred to diffusible hydrogen in this work and vary linearly with hydrogen activity at entry side (see Figure 8). The amount of hydrogen in the material was greatly increased by the cold rolling process from 0.02 – A to 0.66 – CR (IM) wt. ppm (at activity 60). The CR (AM) sample was cast from the same 5 wt.% Ni ferritic steel through arc melting (AM) instead of induction melting process (IM). Through this way, the content of impurity oxides was greatly minimized. The cold rolling process for both CR (IM) and CR (AM) steels is the same. It can be seen in Figure 7 that CR (AM) has a slightly lower hydrogen amount (0.56 wt. ppm) than CR (IM). This difference is attributed to the elimination of impurity oxide-induced defects. CR (IM) – 300°C for 24 h was obtained by heating CR (IM) sample at 300°C in air for 24 h. Annealing in this temperature range (200–300°C) is usually called recovery. This temperature range is not high enough for recrystallization, which occurs at about 0.4 of melting temperature (*T*_m_) of the material [60]. Hence, a distinctive feature of the recovery process is that it does not involve any changes in the grain structure of the cold worked material. Most point defects (such as vacancies) and residue stress field, however, will be eliminated during recovery. Some dislocation rearrangements will occur within existing grains, which, however, contribute only to very tiny amount of total dislocation densities. Therefore, after recovery process, the decrease of overall hydrogen amount in CR (IM) – 300°C for 24 h – 0.29 wt. ppm compared to CR (IM) – 0.66 wt. ppm is mainly due to the annihilation mainly of vacancies in agreement with Krieger et al. [25]. The RK (recrystallized) samples were obtained by heating CR (IM) sample at 500°C for 24 h. After the recrystallization process, most strain fields around the dislocations are removed at the boundaries of newly formed grains through thermal activated motion. Figure 7 also shows that most of hydrogen traps are gone after the recrystallization process, since the total concentration of H in RK went down to a value (0.03 wt. ppm at activity 60) close to the one found for A. The slightly higher value of RK (0.03 wt. ppm) than A (0.02 wt. ppm) at activity 60 could partially come from grain boundaries (as the grains are much smaller, thus resulting in much higher amount of grain boundaries). The amount of diffusible hydrogen is proportional to the applied hydrogen activity, at the entry side (hydrogen at deep traps does not change with hydrogen activity), the amount of diffusible hydrogen at activity 1 can be obtained by the heterodyne method (see Figure 8). It can be seen that at a_H_ = 1 the concentration is about 0.00053 wt. ppm which is close to Riecke et al.’s work where they found for diffusible H in α-iron at a_H_ = 1 about 0.00039 wt. ppm [48]. The pie charts in Figure 9 describe how for two different hydrogen activities (10 and 100) hydrogen (in percentage of the total amount) is distributed in each trap site of CR (IM). The denominator for calculating the percentage of hydrogen in each kind of trap shown in the of pie charts is the total hydrogen amount calculated for the CR (IM) sample which is supposed to contain each kind of hydrogen trap shown in pie charts (Figure 9). The ratio of hydrogen trapped in impurity oxide-induced defects is obtained from different hydrogen amount found in CR (IM) and CR (AM) and the ratio of hydrogen trapped in vacancies comes from the difference in total hydrogen amount between CR (IM) and CR (IM) – 300°C for 24 h. Since the recrystallization process eliminates most of deep traps (i.e. vacancies, impurity oxide-induced defects and strain fields around dislocations) in the 5 wt.% Ni steel, the ratio of hydrogen trapped in dislocations is obtained by the different hydrogen amount between CR (IM) and RK with the subtraction of ∆CR (IM)/CR (AM) (impurity oxide-induced defects) and ∆CR (IM)/CR (IM) – 300°C for 24 h (mainly vacancies). The ratio of hydrogen in lattice sites comes from hydrogen amount of A for each hydrogen activity (10 and 100) and the ratio of hydrogen trapped at grain boundaries is derived from the slightly higher hydrogen amount of RK in comparison to A. Since interstitial sites and grain boundaries belong to the shallow traps, the contribution of them will change with different applied hydrogen activity (a_H_) while deep traps do not. It can be also seen that for activities below 100 vacancies are the dominating hydrogen trap sites in 5 wt.% Ni cold-rolled CR (IM) ferritic steel (~60%) followed by dislocations (~20%) and impurity oxide-induced defects (~16%). The latter, however, might not only be located in the oxides or at the interface oxide/matrix, but could also be dislocations and vacancies induced during cold rolling by the presence of the oxides.

### 3.5. An example for hydrogen-induced damage: hydrogen blistering

**Figure 10.**
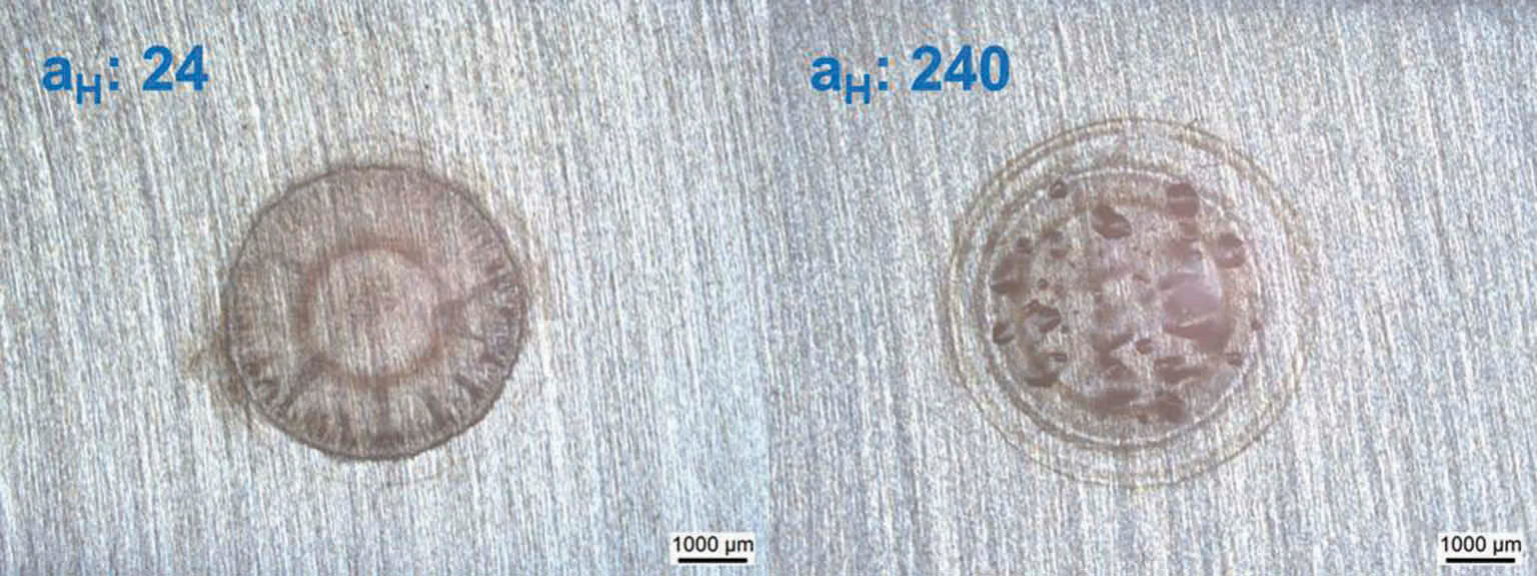
A comparison of CR (IM) steels loaded with different hydrogen activities (a_H_: 24 & 240, assumed same as on pure iron [45]). Left: charged in 0.1 M H_2_SO_4_, galvanostat: −2 mA/cm^2^; Right: charged in 0.1 M H_2_SO_4_ + 2 mM thiourea, galvanostat: −2 mA/cm^2^. Both samples were charged for 100 min.

**Figure 11.**
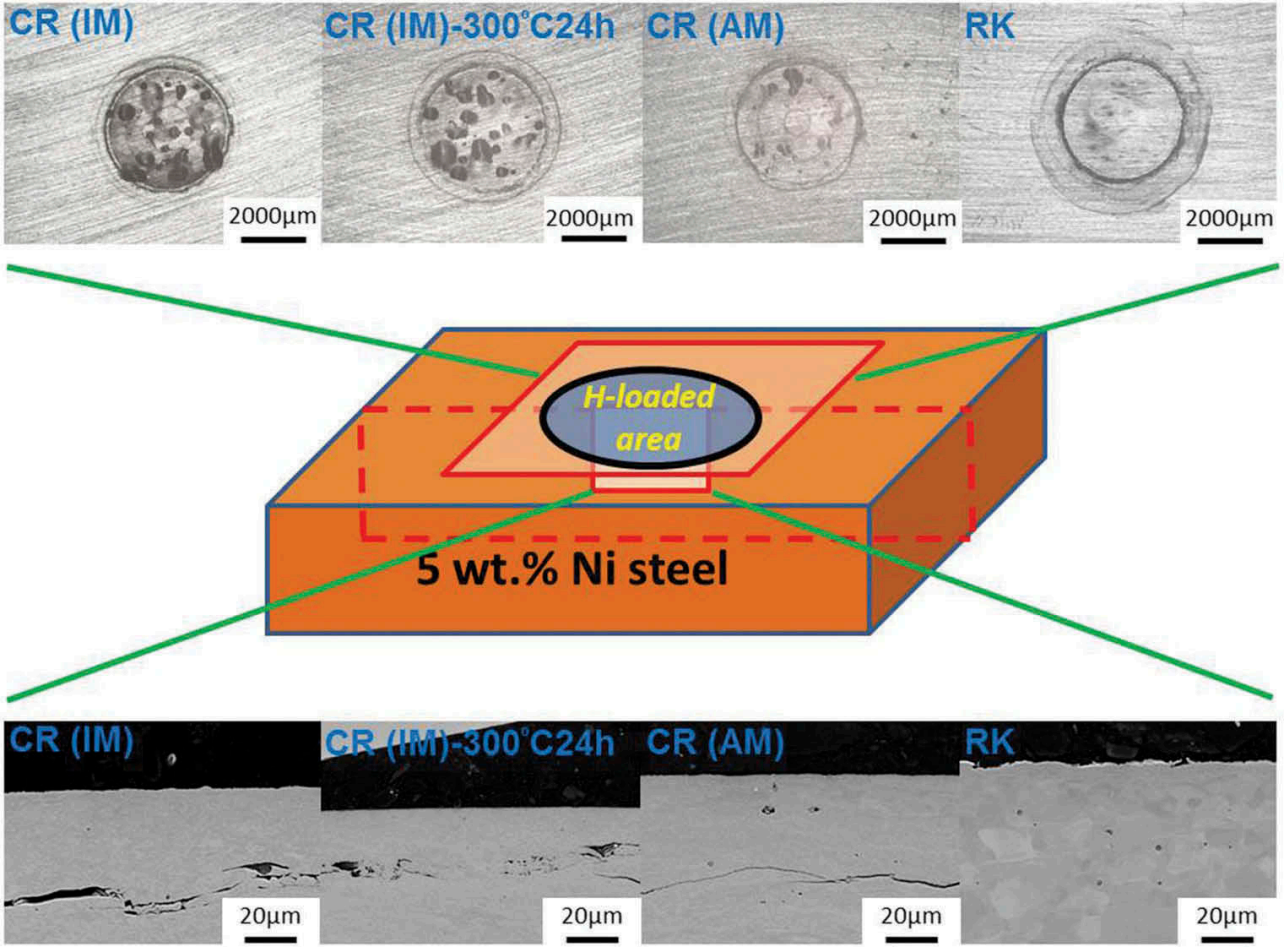
OM surface morphologies and SEM cross-sectional images of different pre-treated 5 wt.% Ni ferritic steels loaded with high hydrogen activity (all steel samples were charged in 0.1 M H_2_SO_4_ + 2 mM thiourea, galvanostatic polarization: cathodic current density *i*_c_ = −2 mA/cm^2^ for 100 min, a_H_: 240, assumed same as on pure iron [45]).

**Figure 12.**
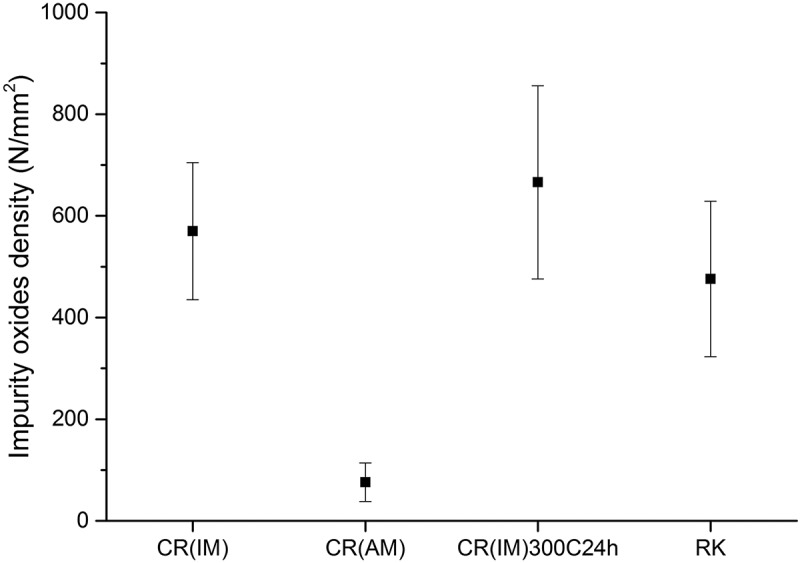
Impurity oxide density (numbers per square meter) of four different pre-treated 5 wt.% Ni ferritic steels.

**Figure 13.**
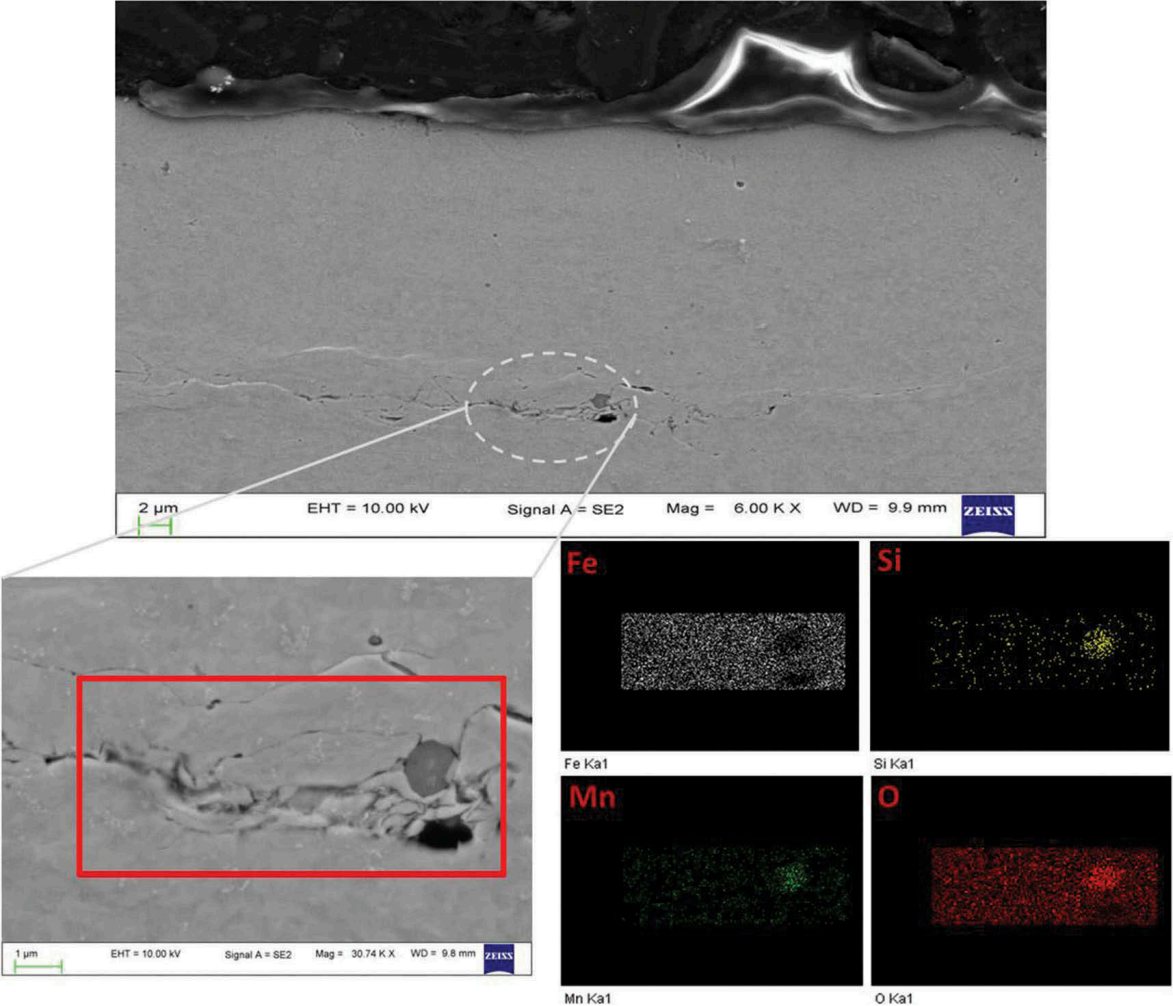
Upper panel: cross-sectional view of CR steel after hydrogen loading (a_H_: 240) for 1 h. Lower panel: dashed circle area in upper graph and EDX elements mapping of the crack initiated region.

As the samples investigated here were partly loaded at extremely high hydrogen activities, it was not surprising that in some cases blistering was observed. Figure 10 shows the effect of different hydrogen activity on the loading side surface. One CR (IM) steel sample (Figure 10, left) was loaded with hydrogen in 0.1 M sulfuric acid without addition of thiourea, and the other CR (IM) steel sample (Figure 10, right) was loaded with hydrogen in 0.1 M sulfuric acid with 2 mM thiourea. The addition of thiourea can inhibit the recombination process of H_2_ molecule and increase hydrogen uptake (hydrogen activity). At the higher H activity of a = 240 the equivalent pressure (equivalent to 5.7 GPa) is much higher than the strength of these materials and blistering is expected. At the lower activity 24 (equiv. to about 57 MPa), no blistering is expected. Figure 11 shows the case of four different pre-treated 5 wt.% Ni ferritic steel samples charged under high hydrogen activity conditions. CR (IM) steel was obtained by an induction melting process while CR (AM) steel was produced by arc melting process from 5 wt.% Ni ferritic alloy. CR(IM) – 300°C for 24 h steel was prepared by heating CR (IM) steel in air at 300°C for 24 h while RK (recrystallized) steel was made by heating CR (IM) steel in air at 500°C for 24 h. It can be observed in Figure 11 that CR (IM) steel has the highest amount of blisters formed on surface. CR (IM) – 300°C for 24 h steel has only slightly lower amount of blisters than CR (IM) steel. For CR (AM) steel, the blister concentration is greatly reduced, which can be clearly seen in OM surface morphologies. Moreover, there is no blister formation on RK steel under the same charging conditions. The same thing is also shown in cross-sectional views (Figure 11). Four samples were cut across the charged area (centered circle region) and then polished for SEM cross-sectional images. Since hydrogen was charged into the steels from the upper surface, the hydrogen concentration was the highest at the upper surface and the lowest at lower surface (a concentration gradient of hydrogen was established inside the steel during charging). That is why most hydrogen-induced cracks were found close to upper surface. Serious cracking damages observed in CR (IM) and CR (IM) – 300°C for 24 h steels are consistent with surface morphologies shown in Figure 10. On the other hand, only slight cracks are observed in CR (AM) steel and no cracks are found in RK steel. It can be seen from Figure 12 that all steel samples produced by induction melting (IM) have more or less similar impurity oxides density (500~700 N/mm^2^), while for steels produced from arc melting (AM) the impurity oxides density is much lower (~70 N/mm^2^). Besides, it can be seen in Figure 13 that the chemical composition of these impurity oxides contains Si and Mn. These two elements did not come from original chemical composition of 5 wt.% Ni ferritic alloy and mostly were contaminations from induction melting (IM) furnace. It can also be seen in Figure 13 that those impurity oxides are acting as initial sites of hydrogen-induced cracking.

## 4. Discussion

### 4.1. Influence of PVD Pd thin film microstructure on permeation results

It was generally reported in the literature that the use of Pd coating in the oxidation cell ensure reliable hydrogen permeation results in Devanathan-Stachurski setup [31,32]. This Pd coating can be either prepared by electrodeposition process or PVD deposition. However, some authors [61–63] criticize that the procedure of Pd coating was usually not documented in the literature, and pointed out defective, porous Pd coating and Fe-Pd interface will trap more hydrogen and affect the permeation results obtained in Devanathan-Stachurski hydrogen permeation cell. In order to study the effect of the sample preparation process of PVD deposited Pd thin film layer on the permeation results obtained by hydrogen electrode method, a comparison of different PVD systems, substrates and surface roughness was performed. The characterization method used here is to measure the correlation of equilibrium potential with hydrogen concentration (the slope is determined). Four sample preparation processes are investigated in this work: different Pd sources (Pd thin film prepared from old and new PVD systems and deposit on same steel substrate), different surface pre-treatments (Pd thin film prepared from same PVD system and deposit on same steel substrates with different polishing process – ground with SiC sand paper P1000 and polished with OP-S 0.025 µm particles), different substrates (Pd thin film prepared from same PVD system and deposit on glass and steel substrate) and also the formation of an additional layer deposition with 200 nm PVD Cu layer before PVD Pd deposition was chosen as an extreme case. The summary of the results is shown in Table 3. It was already reported in Kirchheim et al.’s work [44] that trap sites in Pd samples will cause deviation from theoretical values of −59 mV/decade in the Nernst equation in comparison to annealed Pd sample, which was also found in Evers et al. [37] and is the explanation for the much higher dependence found for the Pd films. These −130 mV/decade in the slope of E vs. log t are now found to be quite robust and not affected too much (only (±10 mV/decade) by grain size, crystal planes of Pd thin film, surface roughness of the substrate (i.e. substrate/Pd interface). Only massive changes such as a copper layer will have an influence on the results obtained by hydrogen electrode-based hydrogen permeation method. −170 mV/decade in the case with 200 nm Cu at steel/Pd interface. Since the microstructure of PVD Pd thin films is similar on steel and copper surface, it is hence assumed here that the formation of new interface (Cu/Pd) creates much more trap sites than original interface (steel/Pd) and leads to abbreviated results. The potential dependence, deviating from the perfect Nernstian dependence of −59 mV/decade of concentration, is due to the presence of traps inside the films and at the interface with the substrate, which causes a deviation between concentration and activity of hydrogen. Concerning lateral resolution for hydrogen detection achievable with these Pd films, one crucial factor that determines lateral resolution is the lateral dimension of the grains in the films, as the grain boundaries operate as traps and hinder fast lateral spreading of hydrogen. The grain sizes were indeed not found to deviate much for the different films, which fits well with the similar potential dependence. Hence, no significant differences are expected in terms of resolution.

### 4.2. Factors to hydrogen blistering damage of 5 wt.% Ni ferritic steel

HB is one type of hydrogen damages, which is caused by accumulation of excessive internal hydrogen pressure at subsurface cavities and voids. When atomic hydrogen diffuse through the material and comes to the surface of voids, it can recombine with each other and form hydrogen gas molecules (H_2_). The formation of H_2_ molecules will increase the internal gas pressure in voids, and once this internal pressure exceeds the critical value (depends on the strength of steel), it will result in permanent deformation of the material (blisters). The level of HB damage is directly related to hydrogen activity on the material surface. Hydrogen may enter the material through gas or aqueous phase, and the increase of hydrogen activity will enhance hydrogen uptake on the material surface. A direct method to avoid HB is to reduce hydrogen activity on steel surface (see Figure 9). However, in some cases, high hydrogen activity is inevitable, especially when steels are exposed to hydrogen sulphide (H_2_S) containing environments (e.g. oil pipelines). H_2_S will react with iron and form iron sulphide (FeS) and H_2_ molecule. FeS then acts as a catalyst to break H_2_ molecule into atomic hydrogens (H). Hence, detailed studies must be performed to know how to avoid HB damage even at high hydrogen activity conditions that could cause internal stress surpassing the critical strength of the material.

The result shows that although the hydrogen amount in vacancies contributes most to the total amount of hydrogen in all kinds of traps (Figure 9) in 5 wt.% Ni cold-rolled CR (IM) ferritic steel (~60%), it did not play the major role in HB damage. This is proven by the nearly equal amount of blisters found on CR (IM) and CR (IM) – 300°C for 24 h steels. Concerning the effect caused by dislocations (~20%) and impurity oxide-induced defects (~16%), the huge decrease of blisters formed on CR (AM) compared to CR (IM) steel indicates a larger influence of impurity oxides than dislocations and vacancies. Many crack initiation sites were indeed found around impurity oxides in the matrix. Obviously, only the oxides in CR(IM), i.e. CR(IM) and CR(IM) – 300°C for 24 h, are dangerous for HB. It is proposed here that in these cases cracks are existing at the interface with the matrix, where H segregates and recombines. Hence, high pH_2_ can quickly build up. It is hence proposed that cracks were nucleated at impurity oxides/matrix interface and later extend horizontally and interconnect. This is in accordance with reports in the literature [64–66]. Interstitial sites and grain boundaries are generally not considered to cause HB, which is consistent with the observation that no blisters are found on recrystallized (RK) steels even at high hydrogen activity.

## 5. Conclusions

We have demonstrated that the KP-based potentiometric hydrogen electrode method provides reliable results of the time lags and permeation rate. Moreover, the calculation of total amount of hydrogen in all kinds of traps in steel from this method compensates for the drawbacks of TDS measurement where the information of diffusible hydrogen was not accessible. On the other hand, TDS results gave useful information of hydrogen in each kind of deep traps and showed consistency with calculation from hydrogen electrode-based method. This work demonstrated the efficiency of combining TDS and KP-based potentiometric methods. It was shown that the contributions of each hydrogen trap to the total amount of hydrogen in all kinds of traps in the steel (i.e. voids, vacancies, dislocations, etc.) for the example of 5 wt.% Ni cold-rolled steel CR (IM) at given hydrogen activity can be elucidated in detail. Concerning the HB damage on 5 wt.% Ni cold-rolled steel CR (IM), although the heat treatment of recrystallization process can totally annihilate the risk of HB, the mechanical strength decreases. It is shown in this study that the most efficient way to avoid HB for cold-rolled steel CR (IM) is to purify the material (remove the impurity oxides), even though hydrogen trapped at impurity oxides makes only small contribution to total amount of hydrogen in the steel.
